# Implementation strategies for large scale quality improvement initiatives in primary care settings: a qualitative assessment

**DOI:** 10.1186/s12875-023-02200-8

**Published:** 2023-11-17

**Authors:** Debora Goetz Goldberg, Constance Owens-Jasey, Sahar Haghighat, Sneha Kavalloor

**Affiliations:** 1 Department of Health Administration and Policy, Affiliate Faculty, Center for Evidence-Based Behavioral Health, Department of Psychology, George Mason University, 4400 University Drive MS IJ3, Fairfax, VA 22030 USA; 2 Department of Sociology and Anthropology, College of Humanities and Social Sciences, George Mason University, 4400 University Drive, 3G5, Fairfax, VA 22030 USA

**Keywords:** Primary care, Quality improvement, Implementation, Evidence-based practice; qualitative research

## Abstract

**Background and objectives:**

The EvidenceNOW: Advancing Heart Health in Primary Care was designed to assist primary care practices in the US in implementing evidence-based practices in cardiovascular care and building capacity for quality improvement. EvidenceNOW, NCT03054090, was registered with ClinicalTrials.gov on 15/02/2017. The goals of this study were to gain a comprehensive understanding of perspectives from research participants and research team members on the value of implementation strategies and factors that influenced the EvidenceNOW initiative in Virginia.

**Methods:**

In 2018, we conducted 25 focus groups with clinicians and staff at participating practices, including 80 physicians, advanced practice clinicians, practice managers and other practice staff. We also conducted face-to-face and telephone interviews with 22 research team members, including lead investigators, practice facilitators, physician expert consultants, and evaluators. We used the integrated-Promoting Action on Research Implementation in the Health Services (i-PARIHS) framework in our qualitative data analysis and organization of themes.

**Results:**

Implementation strategies valued by both practice representatives and research team members included the kick-off event, on-site practice facilitation, and interaction with physician expert consultants. Remote practice facilitation and web-based tools were used less frequently. Contextual factors that influence quality improvement efforts include leadership support, access to resources, previous quality improvement experience, and practice ownership type (independent compared to health system owned). Many clinicians and staff were overwhelmed by day-to-day activities and experience initiative fatigue, which hindered their ability to fully participate in the EvidenceNOW initiative.

**Conclusions:**

This study provides details on how the practice environment plays an essential role in the implementation of evidence-based practices in primary care. Future efforts to improve quality in primary care practices should consider the context and environment of individual practices, with targeted implementation strategies to meet the needs of independent and health system owned practices. Future efforts to improve quality in primary care practices require strategies to address initiative fatigue among clinicians and practice staff. External support for building capacity for quality improvement could help primary care practices implement and sustain evidence-based practices and improve quality of care.

**Trial registration:**

This project was registered with ClinicalTrials.gov on 15/02/2017 and the identifier is NCT03054090.

**Supplementary Information:**

The online version contains supplementary material available at 10.1186/s12875-023-02200-8.

## Background

The EvidenceNOW: Advancing Heart Health in Primary Care was a $112 million effort, funded by the US Agency for Healthcare Research and Quality (AHRQ) between 2015 and 2019, to implement patient-centered outcomes research evidence in more than 1,500 primary care practices across the US [1–5]. The goals of the EvidenceNOW initiative were to (1) assist primary care practices with implementation of the ABCS of Heart Health [6] to promote aspirin use, blood pressure control, cholesterol management, and smoking cessation for high-risk individuals, and (2) build practice capacity for quality improvement by enhancing the use of performance measurement, teamwork, coordination of care activities, and electronic medical records (EMRs) [7–9]. The EvidenceNOW initiative provided external support to primary care practices to build practices’ capacity to implement clinical evidence and to enhance practices’ use of technology, improve collection and analysis of performance data, and connect with community resources [10].

The goal of this study was to obtain a comprehensive understanding of the value of implementation strategies and factors that influenced the EvidenceNOW initiative in Virginia. The current study builds on our previous research [11] by merging qualitative data obtained from research participants and research team members. The study is unique because we have a tremendous amount of qualitative data from multiple sources, which enabled us to gain a comprehensive view of perceptions of EvidenceNOW strategies and factors that influence implementation. The findings from this research point towards critical strategies for future initiatives aimed at implementing evidence-based practices in primary care.

## Methods

There were seven EvidenceNOW regional cooperatives across the U.S. [12] The Virginia EvidenceNOW cooperative included 220 primary care practices across the state, which encompassed independent practices, health system owned practices, and community health centers. The Virginia cooperative provided external support to assist practices with implementing the ABCS of Heart Health and quality improvement activities [13, 14]. External support offered to practices included: a kickoff meeting, web-based resources, and on-site and remote access to practice facilitators and physician expert consultants. The kickoff meeting was an in-person, collaborative learning event that included an introduction to the research team and goals of the project, review of practice facilitation strategies, presentations by subject matter experts, and opportunities to connect with other primary care practices. The web-based resources available to practices included standard of care protocols, checklists, recorded presentations, and webinars. Practice facilitators, who had expertise in health administration and information technology, and physician expert consultants worked with primary care practices to enhance their use of advanced tools in EMR systems, incorporate population health management strategies, and redesign work processes and reimbursement methods.

### Study design

We used qualitative research methods to explore participants’ perspectives of strategies and factors that influenced implementation of the EvidenceNOW initiative [15, 16]. Study protocols for data collection, analysis and reporting were approved by the George Mason University Institutional Review Board in September 2017. All participants provided informed consent prior to data collection and audio recording. We followed the guidelines outlined in the Consolidated Criteria for Reporting Qualitative Research (COREQ) in writing this report of findings [17].

### Sampling and recruitment

We recruited, by email, primary care practices participating in the EvidenceNOW initiative in Virginia and members of the research team. A maximum variation sampling approach [18] was used to recruit a diverse sample of primary care practices based on the following characteristics: date of entry into the EvidenceNOW initiative; practice ownership; practice size (based on the number of patient visits); and the level of engagement with the initiative (assessed by the practice facilitator). A total of 80 physicians, advanced practice clinicians, nurses, practice managers, and other staff involved in the EvidenceNOW initiative in Virginia participated in focus group sessions. A population-based sampling method was used to recruit research team members for in-depth interviews. All research team members who worked on the EvidenceNOW initiative in Virginia in 2017 and 2018 participated in the study, which included 22 academic researchers, project leaders, practice facilitators, expert physician consultants, and quality improvement professionals.

### Data collection

Experienced facilitators from Alan Newman Research (ANR), a consulting firm located in Richmond, Virginia, conducted the focus groups and in-depth interviews. ANR has extensive experience conducting focus groups and interviews with clinicians and medical office personnel and has worked with the research team in the past. Characteristics of practices and research team members that participated in the current study are presented in Table 1.

#### Focus groups with participating practices

Between January 2018 and April 2018, we conducted 25 focus group sessions, [19, 20] each consisting of three to eight practice representatives. Focus groups were chosen as the interview approach to minimize disruption to the practice operations and to encourage discussion between team members. The composition of each focus group consisted of physicians, advanced practice clinicians, practice managers, and other staff within a specific practice participating in the EvidenceNOW initiative in Virginia. We held 21 in-person focus groups at practice offices and four telephone focus groups with clinicians and staff who were not available to meet in-person. Focus groups were held at the practice location for the convenience of participants and were held at lunch or after office hours to minimize disruption to practice operations. Focus groups lasted between 60 and 80 min. Each participant received $150 compensation upon completion of the focus group session. A copy of the focus group discussion guide is available in Supplementary File number 1.

#### Individual interviews with research team members

We conducted in-depth telephone and in-person interviews with members of the research team. The interviews, which lasted between 30 and 45 min, were conducted between January and May 2018. We also conducted three follow up interviews in July and August 2018 to clarify perspectives on various aspects of the EvidenceNOW initiative and to check our interpretation of the qualitative data from interview transcripts. No incentive was given to research team members for participation. A copy of the key informant interview guide is available in Supplementary File number 2.

**Table 1:** Characteristics of Participating Practices and Research Team Members

PRACTICE CHARACTERISTICS (N = 25)	N (%)
***Size***	
1–5 physicians	17 (68)
6–10 physicians	6 (24)
11 or more physicians	2 (8)
***Ownership***	
Independent/other	14 (56)
Not independent (health system/hospital owned)	11 (44)
***Engagement level***	
Actively working on changes	14 (56)
Somewhat moving toward engaging	5 (20)
Unengaged	6 (24)
**RESEARCH TEAM MEMBER CHARACTERISTICS (N = 22)**	**N (%)**
***Project role***	
Leadership Team	7 (31.8)
Practice Facilitator Team	10 (45.4)
Physician Expert Consultant Team	3 (13.6)
Evaluation Team	2 (9.0)
***Gender***	
Female	11 (50.0)
Male	11 (50.0)
***Time on project***	
Entire length of project	18 (81)
Partial length of project	4 (18)

### Data analysis

We used a multidisciplinary team for data analysis to enrich the meaning of findings and draw from different theories and professional fields. Our research team for this study consisted of experts in qualitative research methods, sociology, medicine, public health, implementation science, healthcare management and health informatics.

We incorporated the integrated-Promoting Action on Research Implementation in the Health Services (i-PARIHS) framework into our analytic approach and organization of themes [21]. This framework, described in Table 2, concentrates on four domains: facilitation, recipients, innovation, and context [22]. We chose the i-PARIHS framework because of its unique emphasis on the role of facilitation, which aligns with the practice facilitation strategies used in the EvidenceNOW initiative.

**Table 2:** i-PARIHS Framework Aligned with Components of the EvidenceNOW Initiative ^*^

i-PARIHS Domain	Domain Definition	EvidenceNOW Components
**Facilitation**	An active implementation process of the innovation that involves facilitators and facilitation processes	External support included a kickoff event, practice facilitators, physician expert consultants, and web-based resources
**Innovation**	Evidence-based practices and strategies that are new to an individual or organization	ABCS of Heart Health and practice transformation approaches to adopt and sustain quality improvement efforts
**Recipients**	Stakeholders who are affected by and/or influence implementation	Practice staff and clinicians
**Context**	Various levels of context that can act to enable or constrain implementation	Internal setting includes organizational and individual characteristics External setting includes government programs and regulations and societal expectations

* adapted from Laycock et al. 2018 [22]

Our qualitative data analysis team met on a regular basis over a sixteen-month period and kept detailed notes of emerging themes, coding comparisons, concept diagrams and updates in our coding scheme. We used “a priori codes” that were drawn from our research questions and the i-PARIHS framework, and “inductive codes” that emerged from the data [23]. We used NVivo software for coding transcripts and field notes. In the first and second stage of analysis our research team analyzed transcripts from the in-depth interviews and focus groups separately. The final stage of analysis involved analyzing the entire data set using an immersion/crystallization approach, [24] which involved triangulating the data from both sources. This process resulted in a refined set of integrated themes [25].

## Results

The EvidenceNOW cooperative in Virginia used external support to guide the implementation of quality improvement activities within participating primary care practices. Implementation strategies valued by both practice representatives and research team members included the kick-off event, on-site practice facilitation, and interaction with physician expert consultants. Remote practice facilitation and web-based tools were used less frequently. Contextual factors that influenced quality improvement efforts include leadership support, access to resources, previous quality improvement experience, and organizational climate.

The data supporting the conclusions of this article are included within the article and its additional files. Table 3, Themes by Analytic Stage, lists themes from each data collection method. Table 4, Themes and Supporting Quotes, Organized by i-PARIHS Domain, provides a list of key themes and supporting quotes from both practice representatives and research team members.

**Table 3:** Themes by Analytic Stage

First Stage Practice Focus Groups	Second Stage Research Team Interviews	Third Stage Triangulation of Data Sources
• Kickoff event was valued by participating practices • Some practices valued assistance from practice coaches • More time needed for onsite coaching • Practices desired more interaction with physician expert consultants • Some practices benefited from the EvidenceNOW initiative, while others reported no difference • Practice motivation for participating in EvidenceNOW was to improve quality and clinician wellbeing • More focus needed on improving clinician and staff well-being • Overlap with other programs influenced success of the initiative • Existing systems for data measurement and health information technology influenced project success • Practice ownership (independent vs. health system owned) influenced the level of engagement • Practice clinicians and staff are overwhelmed with demands from taking part in multiple quality improvement initiatives • Requirements from payers and interaction with EHR system adds burden on physicians/practices	**Project Development/ Management** • Compressed project timeline resulted in numerous implementation challenges • Many challenges in recruiting practices for taking part in the initiative • Collaborative research team consists of experts in practice transformation, quality improvement and research • EvidenceNOW Initiative designed to be flexible and to meet the needs of practices • Research team developed an extensive practice improvement toolkit **Intervention** • Successful kickoff event • Practice coaches were in a complicated position because of competing demands • Expert consultants were valued, but underutilized • On-site facilitation seen as more successful than remote facilitation • Research team members had difficulty meeting diverse goals of the EvidenceNOW initiative **Evaluation and Monitoring** • Research team members experienced challenges obtaining and assessing practice data for the evaluation and assessing tailored intervention approaches across practices	**Facilitation** • Kickoff event useful for researchers and participants • Longer on-site facilitation desired • Need for greater interaction with physician expert consultants • Initiative included a well-designed practice improvement toolkit that was underutilized **Innovation** • EvidenceNOW Initiative aligned with practice goals; however, overlapped with other quality improvement efforts • Small, independent practices valued participation in the initiative **Recipients** • Practice participation motivated by a desire to improve quality care and clinician well-being • Ease of approval for changes to the practice based on ownership type (independent vs. health system owned) **Context** • Leadership support is critical for quality improvement activities • Overburdened practices struggled with initiative requirements • Practices exhibited varying levels of quality improvement skills, knowledge, and resources

### Facilitation

#### Kickoff event

##### Successful kickoff event

The kickoff event was successful in gaining buy-in and fostering enthusiasm. Both research team members and participating practices viewed the kickoff event, an onsite collaborative learning event that introduced the EvidenceNOW initiative, as a successful program component. According to the research team, the kickoff event prepared practices to engage in the initiative and produced substantial “buy in.” Practice representatives echoed this viewpoint stating that the kickoff event stirred their “excitement” and “reeled them in” the project because of the informative presentations by “expert practitioners.” Many practice representatives described their enthusiasm about participating in the project, such as the one below.

[We] went to the kick-off and thought, ‘Well, we’re already doing a lot of these measures anyhow… We have to keep our people healthy. It can’t hurt to see what it’s about.’ We went to the kick-off and said, ‘Hey. We can do this.’ And we came back, and it was like, ‘Woohoo! This is exciting.’ We’re pumped up.

Many practice representatives ranked the kickoff event as the “most important” program component and a key factor for their enthusiasm and engagement in the initiative.

### Practice facilitation

#### Onsite practice facilitation valued

A key theme that emerged from the findings was that onsite practice facilitation activities were valued, while remote practice facilitation was considered less useful. Both the practice participants and research team members agreed that on-site practice facilitation was more conducive for quality improvement efforts than remote practice facilitation. Both groups regarded the practice facilitators as “highly qualified”, “credible” and “experienced.” They believed that onsite practice consultant visits helped to “get the ball rolling”, “clarify expectations” and “offer valuable tools for practice improvement.” One practice representative discussed how the facilitator came to the practice and provided the staff with “tons of” suggestions, as described in the below statement.

…we included [the EvidenceNOW] measurements in with what we were doing for [corporate office] because they could cross over. We definitely saw an improvement because [the practice facilitator is] phenomenal about getting tools and different information about how to get patients in, how to get numbers up, what we should be doing.

Practice participants and research team members shared similar views that remote practice facilitation was not utilized to its full potential because practices were less engaged with remote activities than with on-site activities. Multiple practice representatives stated the “timeframe” for the on-site practice facilitation phase, 3 months, was too short and left the practice unprepared for remote practice facilitation. Research team members emphasized a lack of readiness among practices in the transition from on-site practice facilitation to remote facilitation. When asked about what could improve the EvidenceNOW project, many practice representatives made comments such as “more in-person [facilitation]” and “better follow up and support” from facilitators.

### Physician expert consultants

#### More physician expert consultation desired

One strategy for implementation was the inclusion of physician expert consultants to assist practices with quality improvement. All participants, whether part of the research team or participating practices, viewed expert consultants as a “highly valuable resource.” Research team members reported that expert consultants were “underutilized”, and some referred to this as a “missed opportunity.” For many practice representatives, this “missed opportunity” consisted of a lack of knowledge about the availability of expert consultants and how to access this resource, such as the desire from one physician below.

I would have liked a clinical person to say, ‘Yes, we’ve done that in our practice, and this is how you can try to get the providers to, maybe, get along with it.

Research team members thought the lack of awareness among participating practices was the result of miscommunication. Research team leaders described purposefully limiting communication about this resource due to fears of over engaging the physician expert consultants. This concern was described by one member of the leadership team in the quote below:

I was worried that we would get killed by people wanting to talk to our experts and [another member of the leadership team] came up with the idea, ‘Well, just set up office hours. Tell them that [the expert consultant] is going to be available on this date from this time to this time.’ We didn’t, we weren’t killed. They were hardly used.

Several research team leaders stated they were unaware of the extremely low rates of utilization of physician expert consultants during the implementation phase of the initiative.

### Web-based resources

#### Web-based resources underutilized

The web-based resources were well-regarded, although underutilized. Web-based resources offered to participating practices included educational materials, checklists, and webinars. The research team showed pride in the compilation of resources, which was reinforced by practice representatives who confirmed the web-based resources “had a lot of good stuff on there.” Despite praise for the web-based resources, the online platform that housed the EvidenceNOW materials was another resource perceived as underutilized, as described by one member of the research leadership team:

Well, every week we’re working so hard to post some new content and really try to drive engagement, and people just are not logging in and using it.

Representatives from both independent practices and health system owned practices were very vocal about the challenges of using web-based resources during a busy workday. One practice manager declared:

In a busy practice you have to stop to do a webinar. That’s just not going to work.

The web-based resources were another implementation strategy used by the EvidenceNOW cooperative in Virginia that did not result in high use rates among participating practices.

### Context

#### Leadership support

##### Leadership support is critical

Leadership support was critical for engagement in the EvidenceNOW initiative and for meaningful involvement in quality improvement activities. Representatives from small, independent practices described leadership support as motivating engagement, encouraging collaboration, and authorizing workflow changes. Many representatives from health system owned practices described participation in the EvidenceNOW initiative as a requirement from health system leaders, indicating corporate level support for participation. This was described by one health system practice representative:

We spoke to one or two other [health system practices] and I just wanted to get an idea of the top-down order from [corporate office] is that ‘you’re participating’….

Representatives from health system owned practices also described difficulties gaining approval for implementing quality improvement activities for the EvidenceNOW initiative, such as changing patient care processes and extracting EMR data for performance measurement reports. Independent practices, on the other hand, were quick to make decisions on improvement activities and implementation strategies. Practice facilitators attributed the rapid decision making among independent practices to their ability to work directly with practice leaders and those with decision making authority.

### Organizational climate

#### Overburdened providers and staff

A key theme that emerged from interviews with practice representatives and research team members was an awareness that clinicians and staff were overwhelmed with their job duties and responsibilities. Research team members were surprised at the extent of “fatigue” and “burnout” among health care professionals. Many practice representatives mentioned feeling “overtasked” and “overworked,” which kept them from fully engaging with the EvidenceNOW initiative. Representatives from independent practices struggled because they did “not have enough people” to engage in quality improvement efforts. Representatives from health system owned practices also expressed feelings about being overworked. Numerous practice representatives also stated that they suffered from “initiative overload” as a result of participating in various government or organizational initiatives aimed at improving quality or practice efficiency. This was reflected in the statement made by one practice manager:

Honestly, from what the feedback I got from different staff members, providers, things like that, and me too, it’s honestly just another thing to do on top of all of the things we have to do.

As reflected in the above statement, multiple representatives from health system owned practices described their work on the EvidenceNOW initiative as a checklist needing to be completed rather than an opportunity to make improvements.

### Previous experience and existing resources for quality improvement

#### Experience, knowledge and resources

Practices entered the project with varying skills, knowledge, time, and resources to improve their practice. Representatives from health system owned practices described numerous resources available from their corporate office, which strengthened their capacity for quality improvement but left them lacking personal knowledge about quality improvement activities. Representatives from health system owned practices also reported a lack of authority to implement changes to practice processes and procedures for quality improvement. In contrast, representatives from independent practices reported more personal knowledge of quality improvement activities and control of their processes; however, lacked the necessary time, resources, and support, as stated by one primary care physician:

Being a small practice…I don’t have the reserves, whether it’s financial or man hour, that a large organization would have [to implement quality improvement activities].

The ability of participating practices to implement quality improvement activities in the EvidenceNOW initiative in Virginia was influenced by their previous experience with quality improvement efforts, existing resources, leadership support for quality improvement, and authority to execute changes to practice processes and procedures.

**Table 4:** Themes and Supporting Quotes, Organized by i-PARIHS Domain

i-PARIHS Domain	Themes	Supporting Quotes- Participating Practice	Supporting Quotes- Research Team
**Facilitation**			
Kick-Off Event	The kickoff event was successful for gaining buy-in and fostering enthusiasm	“It was good to be around other professionals looking at the same kind of goals. I thought it was a really good motivator… I enjoyed the kickoff session.” (Physician, Independent Practice)	“The kickoff was powerful enough and really cast the vision.” (Physician Expert Consultant)
Practice Facilitation	More onsite practice facilitation activities were desired, remote facilitation was not valued	[The Practice Facilitator] and I would literally get a plan together. He would come out… meet with my staff … and then we would follow up after a week or so… to see who this worked for, how it did work.” (Physician, Independent Practice)	“You’ve got three months to get the buy-in, get them to let you in the door, figure out what’s going on, start to try and implement some things, and then your time is up.” (Practice Facilitator)
Physician Expert Consultants	Expert consultants were valued, but underused	“No, [access to expert consultants] wasn’t shared with us.” (Physician, Health System Practice)	“I was probably one of the few people, physicians, in the state of Virginia who actually had experience and academic background in doing this kind of work, so my overall feeling was that I was very disappointed that I was not able to make the kind of contribution.” (Physician Expert Consultant)
Web-Based Resources	The web-based resources were well-regarded, although underused	“I can’t say that they were actively involved [with web-based resources] … Honestly, they may have viewed it as just another program.” (Administrative Staff, Health System Practice)	“What was positive is that we were able to write a ton of educational material to practices, such as smoking cessation.” (Project Leadership)
**Context**			
Leadership Support	Leadership support is critical	“Our reason from the CEO standpoint, I think, was because it was a good program for the center, and she could see the value. And we were actually restructuring our quality at the time.“ (Administrative Staff, Independent Practice) “I had no choice in the matter to be involved. It was not voluntary.” (Physician, Health System Practice)	“Barriers that practices face in implementing and doing those things, I would say that those barriers are pretty deep and complex. They probably… need more intensive support…” (Project Leadership)
Organizational Climate	Many practices are overwhelmed with day-to-day activities or experience initiative fatigue	“There’s so much they’re trying to inundate [us with]. They just don’t have time to do that much stuff that kind of gets pushed into the practice where they’ve got to focus more on getting their notes done, getting the patients seen, getting the quality of care done… I think it’s just too much.” (Administrative Staff, Independent Practice)	“They’re all burned out and they’re disgruntled and if their environment is a mess then they’re not really in a position to change.“ (Practice Facilitator)
Experience, Skills, and Resources	Previous experience with quality improvement activities, resources, and skills influence practice participation	“We have been focusing on… using best practices to treat diabetes, using best practices to treat hypertension. So, I’m not sure if it’s the [EvidenceNOW initiative] or… the other initiatives that we’ve been a part of, that’s made the difference.“ (Physician, Independent Practice)	“I would say in most circumstances, there was already some work being done, just in general, but not necessarily a specific focus on those things.“ (Practice Facilitator)

## Discussion

The goals of the EvidenceNOW initiative were to implement evidence-based practices for cardiovascular care and strengthen quality improvement in primary care practices. Recent studies found the EvidenceNOW cooperatives improved cardiovascular prevention among participating practices [26, 27]. Our study revealed key implementation strategies used in the EvidenceNOW cooperative in Virginia, which included the kick-off event, on-site practice facilitation, and physician expert consultants. Our study findings align with previous research on the importance of organizational and environmental context on the adoption of innovations [28–32]. Contextual factors that influenced implementation of the EvidenceNOW initiative in Virginia include leadership support, authority to make changes, access to resources, previous experience with quality improvement, and organizational climate. Future large-scale initiatives to implement evidence-based practices in primary care should consider incorporating kick-off events, on-site practice facilitation, access to physician expert consultants, and limited web-based materials.

One interesting finding was the value practice representatives placed on onsite practice facilitation and their modest use of remote tools and resources. The preference for onsite practice facilitation, however, may have shifted due to the increased use of web-based tools and video conferencing technologies during the coronavirus disease of 2019 (COVID-19) pandemic, which may have strengthened individual knowledge and comfort using remote tools for facilitation. Future research should evaluate implementation approaches that use remote technologies such as video conferencing for practice facilitation.

Our study supports previous research that found clinicians and other practice staff are overwhelmed with the day-to-day responsibilities of patient care and administrative tasks, which may contribute to delays in implementation of evidence-based practices, tension within the organization, and resistance to change [33]. Future large-scale improvement efforts in primary care should include strategies to address workload challenges experienced by clinicians and staff. The limitations of our study include a lack of data collection on the quality of implementation, sustainability of the intervention, and long-term outcomes of the EvidenceNOW initiative. Future quality improvement initiatives in primary care should evaluate implementation quality, intervention sustainability, and long-term practice and patient outcomes [34].

## Conclusion

The EvidenceNOW initiative offered primary care practices a series of external support resources to aid implementation of evidence-based practices in cardiovascular care and quality improvement activities. Our qualitative assessment found that the kickoff event, onsite practice facilitation, and physician expert consultation were valuable implementation strategies to both research team members and members of participating practices. Continued external support for primary care practices will be needed for future implementation of evidence-based practices and advancements in technology. Future large-scale quality improvement initiatives should consider hosting an on-site kick-off event, and provide practices with on-site practice facilitation, access to expert physician consultants, and limited web-based resources.

### Electronic supplementary material

Below is the link to the electronic supplementary material.

Supplementary Material 1

Supplementary Material 2

## Data Availability

Data is provided in the manuscript in the form of quotes within the text and in tables. Additional qualitative data is available from the corresponding author upon request.

## Abbreviations

AHRQ: Agency for Healthcare Research and Quality
ANR: Alan Newman Research
COREQ: Consolidated Criteria for Reporting Qualitative Research
COVID-19: Coronavirus disease of 2019
EMR: Electronic Medical Record
i-PARIHS: Integrated-Promoting Action on Research Implementation in the Health Services
