# Supplementary material for: Implementation strategies for large scale quality improvement initiatives in primary care settings: a qualitative assessment
Source: BMC Prim Care. 2023 Nov 17;24:242. doi: 10.1186/s12875-023-02200-8 (PMC10655333; doi:10.1186/s12875-023-02200-8)
Supplement: Supplementary file 1 — Supplementary Material 1 [file 12875_2023_2200_MOESM1_ESM.docx]

***EvidenceNOW Heart of Virginia Healthcare (HVH) Initiative***

**Key Informant Interviews**

**Interview Guide**

**I. INTRODUCTION (5 minutes)**

1. Purpose. As you know, George Mason University (GMU) has been engaged to conduct an evaluation of the *Heart of Virginia Healthcare (HVH)* initiative. We are reaching out to researchers, coaches, project leadership and staff, and participating practices to understand various viewpoints on the development, implementation, outcomes, and sustainability of the project. These interviews are the last stage of data collection for the evaluation effort.

Your feedback, in combination with that of other participants, will be used to help GMU critically examine the experience and opinions of those who participated in the Heart of Virginia Healthcare (HVH) initiative. It may also help to inform future initiatives offered through the Agency for Healthcare Research and Quality (AHRQ).

B. Disclosure

- Observation (*if applicable*)
- Audio recording – For research purposes only
- Findings and comments about the research topics will be summarized collectively across research participants; they will NOT be attributed by name to any specific individuals or to specific practices.

C. Participant Introduction

- Name, title, organization, time on project (in months/years), role on HVH project.

**II. OVERALL INITIATIVE EXPERIENCE (5 minutes)**

1. History of involvement and experience. This part of the analysis will include descriptions of your experience with the Heart of Virginia initiative.

- What were your objectives for participating in the HVH project? What activities did you work on as part of the HVH initiative? What would you describe as your main accomplishments on the project?
- Were there activities or goals you had for the HVH project that were not accomplished? (If yes) Can you describe these activities or goals? What prevented these from being accomplished?

**III. EVALUATION OF INITIATIVE APPROACH AND FEATURES (30 minutes)**

.

1. Reflective: Following is a series of questions about your perceptions on the outcomes of the HVH project. Please provide a context for your perceptions by describing examples from your experience.

- Based on your experiences so far, what outcomes or effects has the Heart of Virginia Healthcare program had? We are interested in positive outcomes or consequences, as well as any negative outcomes.

Additional probes if not specifically addressed:

- Do you believe the HVH initiative has reduced healthcare costs. Why or why not?
- Do you believe the HVH initiative has changed practice patterns. Why or why not?
- Do you believe the HVH initiative has changed the way primary care practice operates? Why or why not?
- Do you believe the HVH initiative has encouraged teamwork within practices that participated? Between practices that participated and other organizations, such as other providers?
- Do you believe the HVH initiative encouraged evidence-based treatment? Why or why not?
- Do you believe the HVH initiative encouraged the coordination of care for at participating practices? Why or why not?
- Do you believe the HVH initiative decreased burnout among providers in participating practices? Why or why not?

1. ABCS-related Outcomes. Has the initiative influenced attention of practices in VA to cardiovascular disease prevention and the ‘ABCS’ *(If yes, probe to determine why and how practices have changed. If no, why not?)*

- May probe on some or of the following possible changes:
- Approach for identifying patients in need of ABCS
- Delivery of ABCS
- Adopting clinical guidelines for ABCS
- Encouraging access to care for ABCS
- Optimizing visits for ABCS
- Defining measures of quality for ABCS
- Developing care plans for ABCS

1. Strategy for Practice Improvement. *Practices in the research study were allowed to choose from a list of improvement strategies under the categories “The ABCS of Heart Health” and “Supportive Practice Environment.”*
   - - - Were there particular strategies that you thought were effective? Why or why not?
       - Were there particular strategies that you thought were not effective? Why or why not?

- (note to probe: Delivery of the ABCS, identifying patients in need of ABCS, adopting clinical guidelines, access to care, developing care plans, optimizing visits, managing medication, supporting self-management, coordinating care, optimizing teamwork, work flow, clinical data and financial picture).
- What would you do differently next time?

1. Next, we would like you to discuss specific program features of the *Heart of Virginia Healthcare* initiative. We would like you to please *rate the importance* of several specific features of the *Heart of Virginia Healthcare* initiative. Let’s discuss several features of the program. For each one, I am interested in understanding whether or not you found it to be useful. *Interviewer will explore (1) awareness, (2) perceived pros and cons, (3) suggestions for improvement, and (7) anything else pertinent to share (ease of use, usability suggestions, spreading awareness).*

- Kickoff training event
- Coaching (by HVH coaches)
  - Visits
  - Phone calls
  - Other?
- Expert consultation
- Collaborative learning events or networking with other participating practices
- Online support center
- Webinars
- Email alerts; tweets or other online alerts
- Other

E. Most/Least Valuable Initiative Features.

- What specific features or aspects of the HVH Initiative have you found to be most important and valuable? Why?
- Are there any specific aspects of the HVH initiative that you perceive as negative or not particularly helpful or valuable? Why?

F. What suggestions for improvement do you have, that would make the HVH initiative better?

G. Discuss and probe as time allows:

- Are there other support services that the *Heart of Virginia Healthcare* initiative could provide? What are they? How will they help? Please be as specific as possible.
- *Should there be* collaboration and networking among practices? If so, how do you picture this happening, ideally? How frequently would this be needed and in what scenarios?

1. **CONCLUSION (1-2 minutes)**
2. Check with *participants* for any additional questions (if applicable).
3. Collect questionnaires.
4. Thank and dismiss participants.
