# Supplementary material for: Implementation strategies for large scale quality improvement initiatives in primary care settings: a qualitative assessment
Source: BMC Prim Care. 2023 Nov 17;24:242. doi: 10.1186/s12875-023-02200-8 (PMC10655333; doi:10.1186/s12875-023-02200-8)
Supplement: Supplementary file 2 — Supplementary Material 2 [file 12875_2023_2200_MOESM2_ESM.docx]

***EvidenceNOW Heart of Virginia Healthcare (HVH) Initiative***

**Focus Group Moderator’s Guide**

**I. INTRODUCTION (15 minutes)**

1. Purpose. George Mason University (GMU) has been engaged to conduct an evaluation of the *Heart of Virginia Healthcare (HVH)* initiative. As part of this important research effort, and to obtain an in-depth understanding of the initiative, we are speaking with participating providers. Some of you may have taken part already in one or more surveys developed by our colleagues regarding the initiative over the past several months. Tonight’s discussion is the next stage in the evaluation effort.

The purpose of this focus group session is to obtain feedback regarding the initiative from your point of view as a provider of primary healthcare to patients in Virginia. We are interested not only in your current perceptions and experiences with the initiative, but also how you think it has influenced you and your practice.

Your feedback, in combination with that of other research participants, will be used to help GMU and other interested organizations develop a better understanding of your perceptions of the initiative. It may also help inform future activities offered to practices through *EvidenceNOW*, the Agency for Healthcare Research and Quality (AHRQ), and *Restoring Primary Care in Virginia*.

B. Disclosure

- Observation (*if applicable*)
- Audio recording – For research purposes only
- Findings and comments about the research topics will be summarized collectively across research participants; they will NOT be attributed by name to any specific individuals or to specific practices. I ask that you NOT share or discuss anything we talk about here with others outside of this group.

C. Ground Rules

- Would like to hear from everyone, one at a time.
- Looking for your personal experiences, opinions, and examples.
- Be candid; our moderator does not work for HVH or any government agency and has no vested interest.

1. Participant Introductions and Practice Description

- Name, specialty, years in practice, years associated with the practice you are currently part of.

*Note: For groups in which all providers are from a single practice, the following information will be collected once. For groups in which multiple practices are represented, it will be collected during introductions by practice.*

- Description of practice – Ownership – hospital-owned or independent, size/number of providers, payer mix, (your description of patient population – old, young, etc.)
- EHR system – software brand, years in place
- PCMH – present or past certification
- Prior quality improvement experience (e.g. using the EHR to track and monitor quality outcomes, participation in pay-for-performance programs, etc.)

**II. OVERALL INITIATIVE EVALUATION (45 minutes)**

1. History of involvement. Now we’d like to spend a little time talking about the history of your involvement with the Heart of Virginia initiative.

- Reasons for participation. Why did your practice join the program? What benefits were you hoping to achieve? What potential downsides were you concerned about? (Note: See below for probes if hospital system-based practice)
- Was joining an easy or hard decision to make? If hard, what prompted your practice to go ahead and enroll? (Note: See below for probes if hospital system-based practice)
- Can you describe how your practice was involved in the program? Who at your practice was involved in the program? What role(s) did they play?
- What were your initial experiences with the program like?
- Would you describe your practice as engaged or disengaged with the program?
- Explain why your practice was engaged/disengaged during the initiative.
- *Probe* - What were some of the challenges or barriers to engaging in the program?
- *Probe* – What, if anything, do you think would have improved your practice’s engagement with the program?
- For hospital systems. (If your practice is part of a hospital system) Can you describe how the hospital system was involved in the decision for your practice to participate in this initiative? Did your hospital system provide resources or assistance for participating in this initiative? Does the hospital system provide resources for general quality improvement at the practice level? And if so, can you describe what resources or assistance are provided by the hospital system?
- Supportive Practice Environment. Did your practice have leadership support for participating in program? Was there clinician buy-in for the initiative*? [We know that for some practices the hospital signed them up, but the practice was not enthusiastic. You may need to clarify which “leader” you mean— the practice leader or the hospital owner.]*

1. Impact/outcomes of program participation (unaided). Based on your experiences so far, what outcomes or effects has the *Heart of Virginia Healthcare* program had? We are interested in positive outcomes or consequences, as well as any negative outcomes.

How has it affected:

- You, as a provider, and your practice of medicine: Has it changed your behavior in any way? Are you doing anything differently as a result of the *Heart of Virginia Healthcare* program? What, if anything, has it motivated you to do?
- Your overall practice operations, staffing, or workflow?
- Do you think participating in the *Heart of Virginia Healthcare* program has improved care for your patients?

1. Strategy for Practice Improvement. *Practices in the research study were allowed to choose from a list of improvement strategies under the categories “The ABCS of Heart Health” and “Supportive Practice Environment.”*
   - - - Do you know which strategies your practice chose?

- Considering the list of “VA Strategies,” can you explain why your practice chose specific strategies? Who in the practice was involved in selection of strategies?
- Please describe how your practice adopted the selected strategies.
- What were the barriers for the practice to adopting these specific strategies? Facilitators?
- Which other strategies, if any, would you have liked help with?

E. ABCS-related Impacts. Has the initiative influenced attention of your practice to cardiovascular disease prevention and the ‘ABCS’ *(Aspirin use by high-risk individuals, Blood pressure control, Cholesterol management, and Smoking cessation)*? *(If yes, probe to determine why and how practice has changed. If no, why not?)*

- May probe on some or of the following possible changes:
- Approach for identifying patients in need of ABCS
- Delivery of ABCS
- Adopting clinical guidelines for ABCS
- Encouraging access to care for ABCS
- Optimizing visits for ABCS
- Defining measures of quality for ABCS
- Developing care plans for ABCS

F. Impact on Burnout and Joy in Practice. We have been told that a primary objective of this initiative is to reduce workplace burnout and to ‘restore the joy to practice’ by giving physicians support and tools which allow them to focus on physician-level work.

- Were you aware that this workplace environment objective was part of the Heart of Virginia Healthcare Initiative?
- From your perspective, how did the heart of Virginia Healthcare Initiative address the issues of workplace burnout and restoring joy to the practice?
- Do you feel the initiative has had any impact on your level of burnout or enjoyment in practicing medicine?
  - If yes, how so? If not, why not?
    - Do you feel the initiative *has the potential* to reduce physician burnout or increase satisfaction/enjoyment in practicing medicine? If so, what needs to be done or provided that would make a difference?
  - Beyond this initiative, can you describe what would reduce workplace burnout and improve your enjoyment in practicing medicine?

1. **EVALUATION OF INITIATIVE APPROACH AND FEATURES (30 minutes)**

Next, we would like you to discuss specific program features of the *Heart of Virginia Healthcare* initiative. Before we discuss this as a group, I would like you to please *rate the importance* of several specific features of the *Heart of Virginia Healthcare* initiative. After rating them, please *rank them* from 1 to 11 based on *order of importance to you*, where “1” is “most important” and “11” is “least important.”

1. Completion of Assessment 2 of 2. *Hand out* HVH Initiative Assessment: Rating and Ranking *and have participants complete the survey. Ensure participants have no questions about rating and ranking, and make sure both pages of the survey are completed (both rating and ranking items).*

B. Let’s discuss several features of the program. For each one, I am interested in understanding whether or not you made use of it and, if so, whether or not you found it to be useful. *Moderator will explore (1) awareness, (2) actual use and experiences, (3) frequency of use, (4) how used, (5) perceived pros and cons, (6) suggestions for improvement, and (7) anything else pertinent to share (ease of use, usability suggestions, spreading awareness).*

- Kickoff training event
- Coaching (by HVH coaches)
  - Visits
  - Phone calls
  - Other?
- Expert consultation
- Collaborative learning events or networking with other participating practices
- Online support center
- Webinars
- Email alerts; tweets or other online alerts
- Other

C. Most/Least Valuable Initiative Features.

- What specific features or aspects of the Initiative have you found to be most important and valuable to you and to your patients? Why?
- Did any aspect or feature of the initiative positively impact your performance? Which ones and how?
- Are there any specific aspects of the initiative that you perceive as negative or not particularly helpful or valuable? Why?

D. Thinking about the initiative overall, what information has proven useful to you? In what format(s) or through what channel(s) did you receive this information?

E. What one or two suggestions for improvement do you have, that would make the initiative better for you, as a participating provider? Better for your patients?

F. Discuss and probe as time allows:

- Are there other support services that the *Heart of Virginia Healthcare* initiative could provide that would help you? What are they? How will they help? Please be as specific as possible.
- Does your practice communicate or interact with other practices within the *Heart of Virginia Healthcare* network or within your initiative cohort? Which ones? Why? Through what means or channels and regarding what topics? How often? (If not, why not?) If so, were these interactions helpful for implementing the Initiative or for overall improvement efforts?
- *Should there be* collaboration and networking among practices? If so, how do you picture this happening, ideally? How frequently would this be needed and in what scenarios? How would this be beneficial to you and others at your practice? (If not, why not?)

1. **CONCLUSION (~10 minutes)**
2. Check with *observers* for any additional questions (if applicable).
3. Thank, compensate, and dismiss participants.
